# Q‐Switched 1064‐nm Laser Versus Picosecond 1064‐nm Laser in Tattoo Removal: Comparable Overall Effectiveness With Higher Excellent Clearance Rates and Fewer Treatment Sessions for the Picosecond Laser

**DOI:** 10.1111/jocd.71098

**Published:** 2026-08-01

**Authors:** Jiaoquan Chen, Yingxue Liu, Yeqing Gong, Shaoyin Ma, Bihua Liang, Huilan Zhu

**Affiliations:** ^1^ Department of Physical Therapy Guangzhou Dermatology Hospital Guangzhou Guangdong China

**Keywords:** efficacy, picosecond laser, Q‐switched laser, safety, tattoo removal

## Abstract

**Background:**

Laser tattoo removal is becoming more popular, with the Q‐switched (QS) 1064‐nm nanosecond laser considered the traditional standard. While the picosecond (PS) 1064‐nm laser offers an alternative for optimizing treatment protocols, comparative data regarding its relative efficacy remain limited and inconsistent.

**Aim:**

This study aims to evaluate and compare the clinical efficacy and safety profiles of QS 1064‐nm laser and PS 1064‐nm laser for tattoo removal.

**Methods:**

A retrospective analysis was conducted on 130 patients with tattoos between January 2015 and September 2024 in a hospital. Participants received either QS 1064‐nm laser (*n* = 57) or PS 1064‐nm laser (*n* = 73) therapy. Efficacy was measured utilizing a four‐grade clearance scale (poor to excellent), and adverse effects were recorded.

**Results:**

The PS 1064‐nm laser achieved significantly higher excellent clearance (78.1% vs. 54.4%, *p* = 0.029) and required fewer median sessions (3 vs. 4, *p* = 0.020). Although the overall effectiveness rate (> 50% clearance) was higher for the PS 1064‐nm laser (94.52%) than for the QS 1064‐nm laser (85.96%), this trend did not reach statistical significance (*p* = 0.094). After adjusting for baseline color imbalance (*p* = 0.061), a subgroup analysis of blue/black tattoos (*n* = 108) showed PS 1064‐nm laser had significantly greater excellent clearance (80% vs. 55.8%, *p* = 0.007), with comparable overall effectiveness (93.9% vs. 88.4%, *p* = 0.314). Both treatments maintained excellent safety performance; reported side effects were limited to transient erythema/edema, with no severe complications reported.

**Conclusion:**

The PS 1064‐nm laser showed comparable overall effectiveness to the QS 1064‐nm laser, with advantages in excellent clearance and fewer sessions. Limitations include predominantly blue/black tattoos and a 3‐month follow‐up, necessitating prospective validation.

## 1 Introduction

Tattoos, once primarily symbols of cultural identity, have evolved into a prevalent form of contemporary self‐expression. As their popularity has grown, so too has the demand for tattoo removal, driven by shifting aesthetic preferences, workplace requirements, and social pressures [1]. Traditional interventions, such as surgical excision and chemical treatments, have largely been replaced by laser techniques, which offer greater precision, reduced invasiveness, and lower the risk of scarring [2]. For several decades, the Q‐switched (QS) Nd:YAG laser operating at 1064‐nm has served as the clinical standard. This technology utilizes nanosecond (ns) pulses to fragment tattoo pigments through photomechanical disruption [3, 4]. Recently, the advent of the 1064 nm picosecond (PS) laser has further transformed the field. By emitting ultrashort pulses in the picosecond range, this modality enhances photoacoustic energy to more effectively pulverize ink particles while simultaneously minimizing thermal damage to the surrounding dermal tissue [5, 6].

However, current literature regarding the comparative efficacy of QS 1064‐nm and PS 1064‐nm lasers remains contradictory. Some studies report the superior efficacy of PS 1064‐nm laser for stubborn pigments [7], while others indicate comparable outcomes between the two technologies [8]. Moreover, there is a lack of comprehensive data regarding the long‐term safety profiles, particularly regarding complications such as dyspigmentation and raised scars. This knowledge gap hinders the establishment of standardized, evidence‐based clinical guidelines. This present study retrospectively evaluates the effectiveness and safety of QS 1064‐nm and PS 1064‐nm lasers for tattoo removal within a clinical cohort to address these inconsistencies.

## 2 Methods

### 2.1 Patients

This retrospective study analyzed clinical data from 130 patients with tattoos treated at the Hospital between January 2015 and September 2024. Patients were treated utilizing either a QS 1064‐nm laser (MedLite C6, ConBio, USA) or a PS 1064‐nm laser (Syneron‐Candela, Israel). Treatment assignment was determined by device availability and clinician discretion during the study period. The inclusion criteria were as follows: (1) Patients receiving laser treatment for tattoos during the specified period without other concurrent therapies; (2) provision of written informed consent following a detailed explanation of the treatment mechanism, efficacy, and potential adverse effects; (3) availability of the comprehensive clinical records, including standardized pre‐ and posttreatment photographs for each session. The exclusion criteria included the following: (1) Patients with incomplete clinical data or poor‐quality photographic evidence; (2) patients with immunodeficiency, active infectious diseases, and underlying systemic comorbidities such as uncontrolled diabetes, hypertension, or cardiovascular diseases (excluded due to potential impacts on wound healing and increased risk of complications); (3) patients who were pregnant or lactating.

### 2.2 Treatment Protocol

Before the treatment, all patients and their families received a comprehensive briefing on the treatment plan, associated risks, and mitigation measures, and written informed consent was obtained. Topical anesthesia was administered by applying lidocaine cream (Tongfang Pharmaceutical Group Co. Ltd.) to the target area under occlusion with plastic wrap for 1 h. Tattoos were either treated with a QS 1064‐nm laser (pulse duration: 5–10 ns; wavelength: 1064 nm, fluence: 1.0–6.0 J/cm^2^, spot size: 3–5 mm; a repetition rate of 3–5 Hz) or a PS 1064‐nm laser (pulse duration: 450 ps; wavelength: 1064 nm; fluence: 1.0–6.0 J/cm^2^, spot size: 3–5 mm, a repetition rate of 3–5 Hz). The clinical endpoint was set to the whitening (frosting) appearance on the tattoo surface. Treatment sessions were scheduled at intervals of 8–12 weeks. Final assessments for clearance and adverse effects were conducted at a 3‐month follow‐up after the terminal session.

### 2.3 Efficacy and Safety Evaluation

Patient demographics (age, gender, and Fitzpatrick skin type) and tattoo characteristics (type, location, color, and ink density) were recorded. To minimize bias, three dermatologists, blinded to the specific laser modality utilized, evaluated outcomes by reviewing standardized clinical photographs from baseline, interval sessions, and final follow‐up. The tattoo clearance rate was quantified utilizing a quartile grading scale, which divides responses into four distinct groups: poor (0%–25% clearance), moderate (26%–50% clearance), good (51%–75% clearance), and excellent (76%–100% clearance). To assess inter‐rater reliability among the three dermatologists for the four‐grade clearance scale, Fleiss' kappa (κ) was calculated. The overall agreement was κ = 0.784 (95% CI: 0.709–0.859, *p* < 0.01), indicating substantial agreement. The overall efficacy rate was calculated as the proportion of patients achieving > 50% clearance. Safety was monitored by documenting adverse reactions, including erythema, edema, pustules, hemorrhage, vesicles, hypertrophic scars, post‐inflammatory hyperpigmentation, and hypopigmentation.

### 2.4 Statistical Analysis

Continuous variables were analyzed utilizing independent *t*‐tests or Mann–Whitney *U* tests. Categorical variables were compared utilizing chi‐square or Fisher's exact test. To address baseline color imbalance, a subgroup analysis restricted to blue/black tattoos was performed to compare outcomes between the PS and QS groups. All statistical analyses were performed utilizing the Statistical Package for the Social Sciences software (version 26.0). Statistical significance was defined as *p* < 0.05.

## 3 Results

### 3.1 Demographic and Clinical Characteristics

A total of 130 patients were included in this study, with 73 receiving the PS 1064‐nm laser and 57 receiving the QS 1064‐nm laser. Baseline characteristics were comparable between the two cohorts, revealing no statistically significant differences in age, sex, Fitzpatrick skin type, tattoo location, color, ink density, or tattoo type (all *p* > 0.05; Table 1). The mean age of the overall cohort was 25.79 years, and 58.46% of participants were males. Most tattoos were located on the extremities (60.77%), and they were predominantly blue or black (83.08%).

**TABLE 1:** Characteristics of PS 1064‐nm laser and QS 1064‐nm laser in the treatment of 130 patients with tattoos.

Variables	Total (*n* = 130)	P1064 (*n* = 73)	Q1064 (*n* = 57)	*p*
Age, Mean ± SD	25.79 ± 9.44	25.66 ± 9.47	25.96 ± 9.50	0.855
Gender, *n* (%)				
Female	76 (58.46)	45 (61.64)	31 (54.39)	0.405
Male	54 (41.54)	28 (38.36)	26 (45.61)	
Skin type, *n* (%)				
III	41 (31.54)	26 (35.62)	15 (26.32)	0.257
IV	89 (68.46)	47 (64.38)	42 (73.68)	
Location, *n* (%)				
Head/Face	16 (12.31)	8 (10.96)	8 (14.04)	0.400
Trunk	35 (26.92)	23 (31.51)	12 (21.05)	
Extremities	79 (60.77)	42 (57.53)	37 (64.91)	
Color, *n* (%)				
Multicolor	12 (9.23)	3 (4.11)	9 (15.79)	0.061
Gray/Black	10 (7.69)	5 (6.85)	5 (8.77)	
Blue/Black	108 (83.08)	65 (89.04)	43 (75.44)	
Ink density, *n* (%)				
High	13 (10.00)	6 (8.22)	7 (12.28)	0.178
Moderate	35 (26.92)	15 (20.55)	20 (35.09)	
Low	61 (46.92)	38 (52.05)	23 (40.35)	
Minimal	21 (16.15)	14 (19.18)	7 (12.28)	
Tattoo type, *n* (%)				
Cosmetic	14 (10.77)	7 (9.59)	7 (12.28)	0.849
Traumatic	12 (9.23)	6 (8.22)	6 (10.53)	
Amateur	83 (63.85)	49 (67.12)	34 (59.65)	
Professional	21 (16.15)	11 (15.07)	10 (17.54)	

### 3.2 Treatment Efficacy

**FIGURE 1:**
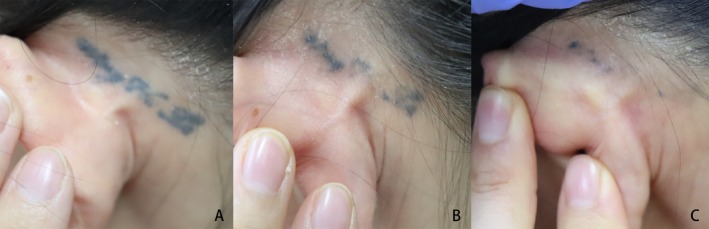
A tattoo behind the left ear treated with the PS 1064‐nm laser (fluence: 1.8–4.3 J/cm^2^, spot size: 3 mm, a repetition rate of 3 Hz). (A) Before treatment, (B) after one treatment, and (C) after two treatments.

**FIGURE 2:**
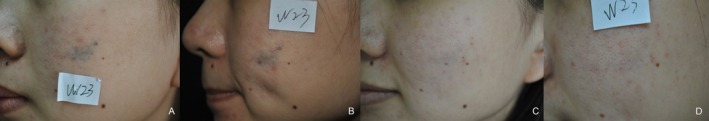
A tattoo on the left cheek treated with the PS 1064‐nm laser (fluence: 2.2–2.4 J/cm^2^, spot size: 3–4 mm, a repetition rate of 3–4 Hz). (A) Before treatment, (B) after one treatment, (C) after two treatments, and (D) after three treatments.

**FIGURE 3:**
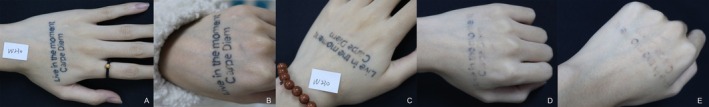
A tattoo on the left back of the hand treated with the PS 1064‐nm laser (fluence: 3.5–5 J/cm^2^, spot size: 3 mm, a repetition rate of 2–3 Hz). (A) Before treatment, (B) after one treatment, (C) after two treatments, (D) after three treatments, and (E) after four treatments.

**FIGURE 4:**
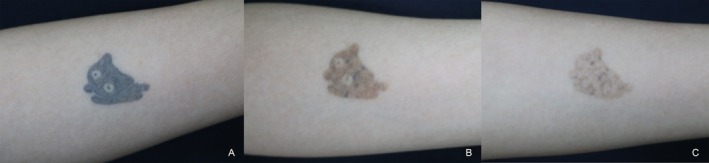
A tattoo on the left forearm treated with the QS 1064‐nm laser (fluence: 3.5–4.5 J/cm^2^, spot size: 3–4 mm, a repetition rate of 2–4 Hz). (A) Before treatment, (B) after one treatment, and (C) after two treatments.

**FIGURE 5:**
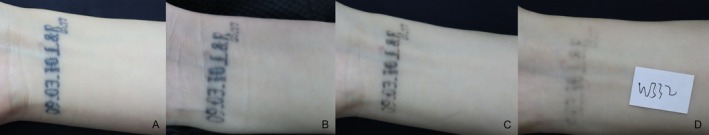
A tattoo on the right forearm treated with the QS 1064‐nm laser (fluence: 3.3–3.9 J/cm^2^, spot size: 3–4 mm, a repetition rate of 3–4 Hz). (A) Before treatment, (B) after one treatment, (C) after two treatments, and (D) after three treatments.

**FIGURE 6:**
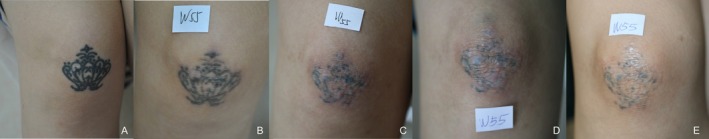
A tattoo on the right knee treated with the QS 1064‐nm laser (fluence: 2.2–4 J/cm^2^, spot size: 3 mm, repetition rate of 3–4 Hz). (A) Before treatment, (B) after one treatment, (C) after two treatments, (D) after three treatments, and (E) after four treatments.

The PS 1064‐nm laser exhibited significantly better performance compared to the QS 1064‐nm laser (Table 2). In the PS 1064‐nm laser cohort, 78.08% (*n* = 57/73) of patients achieved an excellent response (76%–100% clearance) (Figures 1, 2, 3), whereas only 54.39% (*n* = 31/57) reached this threshold in the QS 1064‐nm laser group (*p* = 0.029) (Figures 4, 5, 6). Furthermore, patients in the PS 1064‐nm laser group required significantly fewer median sessions to achieve an excellent response (median: 3.00, IQR: 3.00–4.00) compared to those in the QS 1064‐nm laser group (median: 4.00, IQR: 3.00–5.00; *p* = 0.020). While the overall effectiveness rate (> 50% clearance) was 94.52% (*n* = 69/73) for the PS 1064‐nm laser compared to the QS 1064‐nm laser (85.96%, *n* = 49/57), this difference did not reach statistical significance (*p* = 0.094). However, the median number of sessions required to attain an effective response (> 50% clearance) was significantly lower for the PS 1064‐nm laser (3.00, IQR: 2.00–4.00) than for the QS 1064‐nm laser (4.00, IQR: 2.00–5.00; *p* = 0.034).

**TABLE 2:** Comparison between PS 1064‐nm laser and QS 1064‐nm laser in the treatment of 130 patients with tattoos.

Variables	Total (*n* = 130)	P1064 (*n* = 73)	Q1064 (*n* = 57)	*p*
Efficacy, *n* (%)				
Poor response	4 (3.08)	2 (2.74)	2 (3.51)	**0.029**
Moderate response	8 (6.15)	2 (2.74)	6 (10.53)	
Good response	30 (23.08)	12 (16.44)	18 (31.58)	
Excellent response	88 (67.69)	57 (78.08)	31 (54.39)	
Treatment response, *n* (%)				
Effective	118 (90.77)	69 (94.52)	49 (85.96)	0.094
Ineffective	12 (9.23)	4 (5.48)	8 (14.04)	
Total number of treatments with excellent response				
Range	1–7	1–7	1–6	**0.020**
M (Q_1_, Q_3_)	3.50 (3.00, 4.00)	3.00 (3.00, 4.00)	4.00 (3.00, 5.00)	
Total number of treatments with effective				
Range	1–7	1–7	1–6	**0.034**
M (Q_1_, Q_3_)	3.00 (2.00, 4.00)	3.00 (2.00, 4.00)	4.00 (2.00, 5.00)	

*Note:* Bold values indicate statistically significant differences between the PS 1064‐nm and QS 1064‐nm laser groups (*p* < 0.05).

To address the near‐significant baseline imbalance in tattoo color distribution (blue/black: 89.04% in PS group vs. 75.44% in QS group, *p* = 0.061), we performed a subgroup analysis restricted to patients with blue/black tattoos. Among these 108 patients (65 in PS group, 43 in QS group), the PS 1064‐nm laser achieved an excellent clearance rate of 80% (*n* = 52/65) compared to 55.81% (*n* = 24/43) for the QS 1064‐nm laser (*p* = 0.007). The overall effectiveness rate (> 50% clearance) in the blue/black subgroup was 93.85% (*n* = 61/65) for the PS 1064‐nm laser versus 88.37% (*n* = 38/43) for the QS 1064‐nm laser (*p* = 0.314).

### 3.3 Safety

Both laser modalities maintained excellent safety profiles. Transient adverse effects, such as erythema and edema, were observed in 10.96% (*n* = 8/73) of the PS 1064‐nm laser group and 12.3% (*n* = 7/57) of the QS 1064‐nm laser group. These reactions resolved spontaneously within 48–72 h. In each group, there were two instances of post‐inflammatory hyperpigmentation (PIH). No severe complications, including blistering, scarring, or permanent dyspigmentation, were observed, and no significant intergroup differences in safety were identified (*p* > 0.05).

## 4 Discussion

Tattoo removal has evolved into a widely studied and popular cosmetic procedure, primarily driven by an increasing number of individuals who have tattoos and a tendency to remove them for a variety of personal, social, and professional reasons [1]. Currently, the main techniques used for tattoo removal are based on laser technology, specifically using QS 1064‐nm laser and PS 1064‐nm laser [9]. Most tattoo pigments consist of particles ranging from 30 to 300 nm with relatively shorter thermal relaxation times (< 10 ns) [10]. The 5–10 ns pulse duration of QS 1064‐nm laser approximates this TRT, generating shock waves that rupture the targeted ink particles. Previous studies, such as those by Kilmer et al. [11], reported that 77% (*n* = 30/39) of black tattoos had a great response (more than 75% clearance) after approximately four sessions with QS 1064‐nm laser. Moreover, another study revealed that 95.45% (21 out of 22) of patients had an excellent response after one to four treatments [12]. In contrast, the present cohort achieved an excellent response rate of only 54.39% (*n* = 31/57) after one to six sessions, which is slightly lower than findings from previous studies [13]. This discrepancy might be attributed to the lower fluence used in this research (1–6 J/cm^2^) compared to earlier studies (5–10 J/cm^2^) [12, 13]. Furthermore, patients with darker skin tones (Fitzpatrick types IV–VI) often require more sessions, longer wavelengths, and face a higher risk of adverse effects, particularly hypopigmentation [9]. Although repeated QS laser treatments eventually help reduce tattoo visibility, the treatment efficacy is inconsistent, and therapy duration is extended, which induces substantial distress for both practitioners and patients.

With its ultrashort pulse duration and selective photoacoustic effect, the PS 1064‐nm laser has emerged as a benchmark for tattoo removal [6], offering reduced collateral thermal damage compared to traditional nanosecond lasers. The present investigation revealed that 78.08% (57 out of 73) of patients treated with PS 1064‐nm laser had an excellent response after an average of three sessions. Despite limited comparative data, the present results indicated that the PS laser yields superior outcomes in high‐tier clearance outcomes. This aligns with the findings of Ross et al. [7], Lorgeou et al. [14], and Choi et al. [15], but contradicts the findings of Pinto et al. [8] and Du et al. [16], who reported no significant differences between the two laser types. In the study by Du et al. [16], rats were subjected to a single laser treatment, while in the research by Pinto et al. [8], the cohort experienced a maximum of two sessions. Participants in studies by Ross et al. [7], Lorgeou et al. [14], and our studies received between four and seven treatments. This variation suggests that the PS 1064‐nm laser has more advantages when the number of treatment sessions increases. Moreover, there might be differences in the fluence levels that could explain the inconsistent results. In the study by Du et al. [16], rats with treatment using PS 1064‐nm laser were given a lower fluence (2.0 J/cm^2^) compared to QS 1064‐nm laser, which was set at 4.0–5.0 J/cm^2^ accordingly, the efficacy between the two was comparable. Conversely, another animal study with guinea pigs demonstrated that the PS 1064‐nm laser (3.0 J/cm^2^) performed better than the QS 1064‐nm laser (3.4 J/cm^2^) at comparable fluence levels [15], which supports these observations. Therefore, the effectiveness of these lasers in tattoo removal depends on both the number of treatment sessions and the energy fluence, with the PS 1064‐nm laser requiring fewer sessions and lower energy input to achieve the best results.

Both modalities exhibited excellent safety for Fitzpatrick skin types (predominantly III–IV) in our cohort. Transient erythema and edema were observed without permanent dyspigmentation. Our findings align with those of Cannarozzo et al. [17], who reported that Q‐switched 1064 nm picosecond laser treatment for benign hypermelanosis in Asian patients (Fitzpatrick types II–IV) was well tolerated, with only temporary perilesional erythema and edema that resolved within 1–3 days, and no significant adverse events such as persistent dyspigmentation or scarring. The authors emphasized that careful fluence titration is key to minimizing complications in darker skin tones. Together, these observations suggest that historical concerns regarding laser‐induced hypopigmentation in darker skin tones, which may be mitigated by optimized fluence settings and the 1064 nm wavelength's deeper penetration, which minimizes epidermal damage [15, 18].

Several key limitations of this study must be addressed. First, the retrospective, non‐randomized design introduces potential selection bias, as treatment allocation was based on device availability rather than strict randomization. This fundamental confounder means that patients in the two groups were inherently different, and unmeasured factors—such as differences in tattoo ink composition, particle size, and depth—could have influenced the outcome [9, 10]. The near‐significant baseline imbalance in tattoo color (blue/black: 89.04% in PS group vs. 75.44% in QS group, *p* = 0.061) is particularly concerning, as color independently drives clearance. While we performed a subgroup analysis restricted to blue/black tattoos, a multivariable analysis would be preferable but is limited by sample size and the retrospective design. Second, the predominance of blue/black tattoos limits the applicability of our conclusions to polychromatic tattoos. Third, the 9‐year study period (2015–2024) introduces the possibility of platform and parameter drift, including potential variations in laser handpiece performance, energy calibration, and operator technique evolution over time. Although all treatments adhered to institutional protocols, we were unable to quantify the magnitude of such drift in this retrospective analysis. This temporal heterogeneity may have introduced noise into our efficacy comparisons and should be considered when interpreting the results. Fourth, the 3‐month follow‐up period may be insufficient to capture long‐term recurrence and side effects. Given these constraints, the difference in the overall effectiveness rate (> 50% clearance) lacking statistical significance (94.52% versus 85.96%, *p* = 0.094) further underscores that the perceived superiority of the PS 1064‐nm laser in achieving “excellent” clearance should not be overstated. The findings should be interpreted as generating a hypothesis within the constraints of a retrospective comparison, rather than as definitive evidence of superiority.

The PS 1064‐nm laser demonstrated comparable overall effectiveness to the QS 1064‐nm laser for tattoo removal, with potential advantages in achieving excellent clearance and reducing the number of treatment sessions. Given the retrospective design, non‐randomized allocation, and the nonsignificant difference in overall effectiveness, these findings require validation through prospective, randomized controlled trials.

## Author Contributions

J.C. and Y.L. conceived and designed the study, as well as wrote the manuscript. Y.G., S.M. and B.L. provided study materials and patient data. J.C. and Y.L. collected and assembled the data. J.C. and Y.L. conducted data analysis and wrote the results. B.L. and H.Z. was responsible for reviewing and revising the manuscript.

## Funding

This work was supported by Guangzhou Municipal Science and Technology Program (Grant no. 2023A03J0470 and 2023A03J0943).

## Disclosure

Declaration of Generative AI and AI‐ assisted technologies in the writing process: AI tools (e.g., DeepL) were used to assist with translation and linguistic refinement. The authors reviewed and edited the translated text to ensure accuracy and scientific integrity. All scientific content and analyses were conducted and verified by all authors.

## Ethics Statement

The study protocol was approved by the Ethics Committee of the GuangZhou Dermatology Hospital (gzsp202537). Patients provided written informed consent for the use of their anonymized photographs for research and publication purposes.

## Consent

Patients signed informed consent regarding the publishing of their data and photographs.

## Conflicts of Interest

The authors declare no conflicts of interest.

## Data Availability

The data that support the findings of this study are available from the corresponding author upon reasonable request.
